# Cardiomyocytes in Young Infants With Congenital Heart Disease: a Three-Month Window of Proliferation

**DOI:** 10.1038/srep23188

**Published:** 2016-03-15

**Authors:** Lincai Ye, Lisheng Qiu, Haibo Zhang, Huiwen Chen, Chuan Jiang, Haifa Hong, Jinfen Liu

**Affiliations:** 1 Department of Thoracic and Cardiovascular Surgery, Shanghai Children’s Medical Center, Shanghai Jiaotong University School of Medicine, Shanghai, China; 2 Institute of Pediatric Translational Medicine, Shanghai Children’s Medical Center, Shanghai Jiaotong University School of Medicine, Shanghai, China; 3 Shanghai Institute of PediatricCongenital Heart Disease, Shanghai Children’s Medical Center, Shanghai Jiaotong University School of Medicine, Shanghai, China

## Abstract

Perinatal reduction in cardiomyocyte cell cycle activity is well established in animal models and humans. However, cardiomyocyte cell cycle activity in infants with congenital heart disease (CHD) is unknown, and may provide important information to improve treatment. Human right atrial specimens were obtained from infants during routine surgery to repair ventricular septal defects. The specimens were divided into three groups: group A (age 1–3 months); group B (age, 4–6 months); and group C (age 7–12 months). A dramatic fall in the number of Ki67 -positive CHD cardiac myocytes occurred after three months. When cultured *in vitro*, young CHD myocytes (≤3 months) showed more abundant Ki67-positive cardiomyocytes and greater incorporation of EdU, indicating enhanced proliferation. YAP1 and NICD—important transcript factors in cardiomyocyte development and proliferation—decreased with age and β-catenin increased with age. Compared with those of older infants, cardiomyocytes of young CHD infants (≤3 months) have a higher proliferating capacity *in vivo* and *in vitro*. From the perspective of cardiac muscle regeneration, CHD treatment at a younger age (≤3 months) may be more optimal.

Congenital heart disease (CHD) is the leading cause of birth defect-related morbidity and mortality^1^,^2^, with 48.5% of cardiovascular deaths in children caused by CHD^3^. Although simple CHD (e.g. ventricular septal defect) can be treated completely, complex CHD requires surgical intervention in early infancy and has a high mortality rate^4^,^5^. The first year of life is critical for postnatal heart development. During this period, the structure and function of the infant heart change rapidly, with the heart rate declining from 150 beats per minute to 80 beats per minute and the systolic blood pressure rising from 55 mmHg to 100 mmHg^6^. Almost 55.3% of CHD deaths occur in the first year of life^3^. Although cardiomyocyte cell cycle activity is critical for heart development and damage repair, information on this process is limited. For example, data supporting that the average percentage of M-phase cardiomyocytes is 0.012 ± 0.003% is obtained from two samples only^7^.

Culturing human cardiomyocytes isolated from surgical biopsies is a difficult process, but it is important for translational medical therapy and research. Few studies have successfully used heart tissues from older patients^8^,^9^,^10^. Because infant cardiomyocytes are more resistant to hypoxia, isolating and culturing infant cardiomyocytes may be easier and more useful compared with isolating and culturing older cardiomyocytes. No studies have found markers of proliferation, such as Ki67, or incorporation of 5-Ethynyl-2′-deoxyuridine (EdU) in primary cultured human cardiomyocytes. It is unclear whether isolated cultured young CHD cardiomyocytes sustain proliferative ability.

YAP1, β-catenin, and Notch Intracellular Domain (NICD) are three important transcription factors involved in cardiac development and regeneration, aberrancies of which contribute to CHD development^11^,^12^,^13^,^14^. However, their expression in infant hearts remains mostly unknown. In the present study, we investigated the proliferation of atrial cardiomyocytes in human infants with the most common CHD–ventricular septal defect–and found that cardiomyocytes from young CHD infants (≤3 months) are notably proliferative. We isolated and cultured these CHD cardiomyocytes and learned that younger CHD cardiomyocytes (≤3 months) are easier to culture. We found that the expressions of YAP1, β-catenin, and NICD are conserved in CHD heart tissues compared with mice heart.

## Results

### Patient baseline information and morphological characteristics of atrial cardiomyocytes

Because of the small size of tissues, it was impossible to perform all six desired experiments using a single piece of tissue. Instead, 111 pieces of atrial tissues were collected from children with isolated ventricular septal defect. For each experiment, the tissues were carefully divided into three groups: group A (age 1–3 months); group B (age 4–6 months); and group C (age 7–12 months) (Supplemental Figure S2A). H&E staining to examine the morphology of atrial cardiomyocytes revealed that the average size of cells increased with age (Supplemental Figure S2B), which is consistent with previous reports^15^.

### Young CHD cardiomyocytes (≤3 months) demonstrated higher proliferation rate *in vivo*

**Figure 1:**
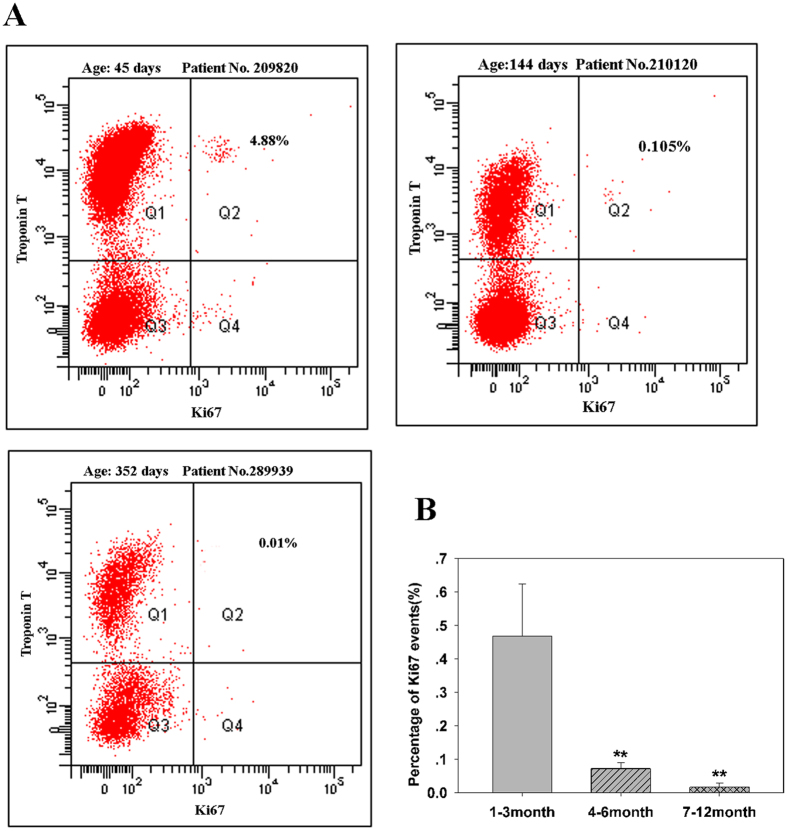
Percentage of Ki67-positive cardiomyocytes by flowcytometry. (**A**) Representative flowcytometry data showing Ki67-positive cardiomyocytes at three different ages; (**B**) Quantification of Ki67-positive cardiomyocytes based on age (n = 6 per age group). Error bars represent SD. *P < 0.05.

**Figure 2:**
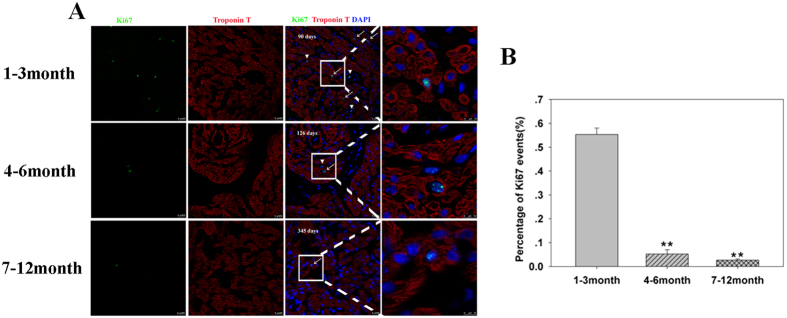
Confocal microscopy of Ki67-positive cardiomyocytes in tissue sections. (**A**) Representative Ki67-positive cardiomyocytes from different age groups. Cardiac Troponin T (red), Ki67 (green), and DAPI (blue). (**B**) Quantification of Ki67-positive cardiomyocytes in three age groups (n = 6 per age group). Error bars represent SD. **P < 0.01.

Flowcytometry analysis is a reliable method of counting rare proliferating cardiomyocytes. As shown in Fig. 1, the percentages and standard deviations of Ki67-positive cardiomyocytes in the three age groups were 0.47 ± 0.16, 0.07 ± 0.02, and 0.02 ± 0.01, respectively (p < 0.01, n = 6 in each group). To confirm these results, the number of proliferating cardiomyocytes was determined using confocal microscopy of tissue sections. The relative numbers of Ki67-positive cardiomyocytes as assessed by this method in the three groups were 0.55 ± 0.02, 0.05 ± 0.01, and 0.02 ± 0.005 (p < 0.01, n = 6, Fig. 2), respectively. These values were in agreement with the results of the flowcytometry analysis. Overall, these results demonstrate that younger CHD cardiomyocytes (aged ≤3 months) have prominent cell cycle activity, which may be manipulated to improve CHD surgery.

### Proliferative ability of young CHD cardiomyocytes (≤3 months) *in vitro*

Human cardiomyocyte isolation from surgical biopsies is not easy, and previous reports showed that the yield of viable cells is 20% at best^8^. We obtained 50–70% viable cardiomyocytes from the young CHD group (Supplemental Fig S3A). There were a greater number of round and small cardiomyocytes in group A compared with those in the other groups (Supplemental Fig S3B and C). Previous reports showed that healthy cardiomyocytes are rod-shaped and dead cardiomyocytes are round^8^, which was true in our results for isolated ventricular cardiomyocytes, but not true for isolated atrial cardiomyocytes (Supplemental Figure S4).

**Figure 3:**
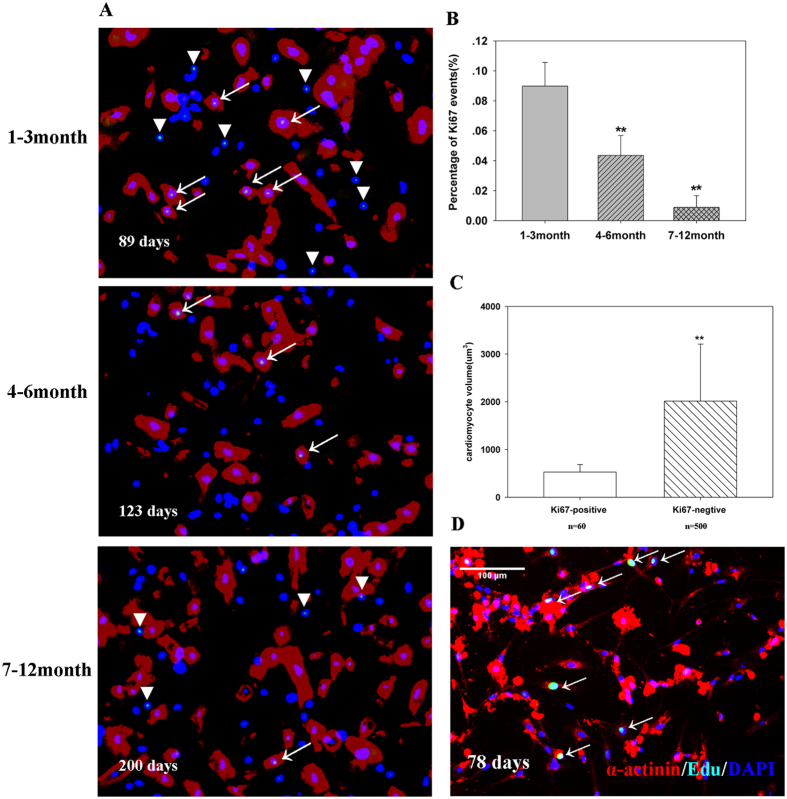
*In vitro* culture of cardiomyocytes. (**A**) Representative graphs of different age groups. Cardiac Troponin T (red), Ki67 (green), and DAPI (blue). Magnification: x10. Arrow indicates proliferating cardiomyocytes, and the triangle indicates non-cardiomyocytes. (**B**) Quantification of Ki67-positive cardiomyocytes (n = 6). Error bars represent SD. **P < 0.01. (**C**) The volume of Ki67-positive cardiomyocytes. P < 0.01 (**D**) After four days of culture, human cardiomyocytes still showed cell cycle activity. Sarcomeric Alpha Actinin (red), EdU (green), and DAPI (blue).

All three groups of cardiomyocytes retained the ability to proliferate *in vitro* (Fig. 3A,B). Ki67-positive cells are relatively smaller in size, with an average size of 529 ± 156 μm^3^ (Fig. 3C). After four days of culture, cardiomyocytes became flat but retained their cell cycle activity (Fig. 3D). These results proved that young CHD cardiomyocytes (≤3 months) are easier to culture and proliferate *in vitro*.

### Expression of YAP1, β-catenin, and NICD in CHD heart tissues

**Figure 4:**
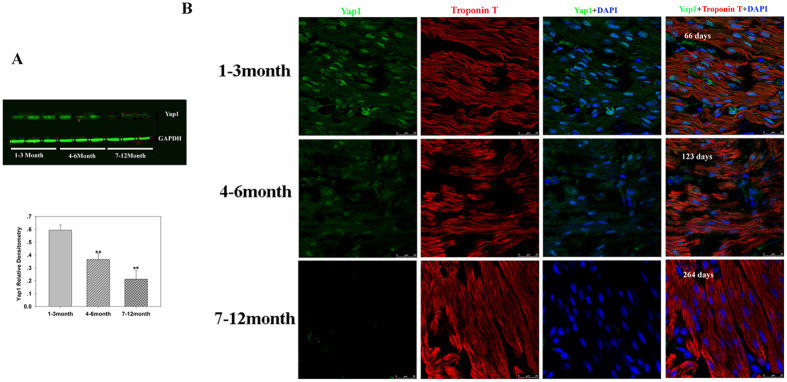
Yap1 expression by Western blot analysis and YAP1 immunofluorescence in tissue sections. (**A**) Representative Western blot (top) and mean data obtained by densitometry analysis (bottom) are shown. GAPDH served as a loading control. The bars indicate mean ± SD. ANOVA was performed to evaluate statistical significance of differences, n = 6, **P < 0.01. (**B**) Representative immunofluorescence in different age groups. Scale bar: 25 μm.

Previous studies on animal models have suggested that YAP1, β-catenin, and NICD tightly control the regenerative ability of animal heart tissue^11^,^12^,^13^,^14^. We investigated whether their expression correlated with CHD cardiomyocyte cell cycle activity to determine which factors require further investigation. Western blotting showed that the levels of YAP1 protein decreased with age (p < 0.05, n = 6, Fig. 4A), which is consistent with von Gisea’s report that the expression of YAP1 in cardiomyocytes and non-cardiomyocytes decreased with mouse heart developmental age^16^. To confirm western blot results, we performed immunofluorescence to visualize the expression of YAP1. The expression of YAP1 protein in cardiomyocytes decreased with age. YAP1 was also expressed in other types of cells (Fig. 4B).

**Figure 5:**
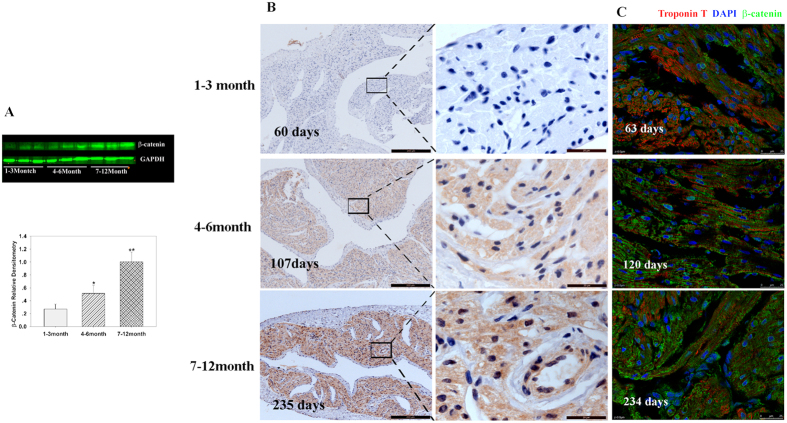
β-catenin expression in cardiomyocytes. (**A**) Representative Western blot analysis (top) and mean data obtained by densitometry analysis (bottom) are shown. GAPDH served as loading control. The bars indicate mean ± SD. ANOVA was performed to evaluate statistical significance of differences, n = 6, **P < 0.01. (**B**) Representative immunohistochemistry in different age groups. Scale bar: Left panel, 100 μm; Right panel, 20 μm. (**C**) Representative immunofluorescence in different age group. Scale bar: 25 μm.

Western blotting was used to investigate the correlation between β-catenin expression and the proliferative ability of cardiomyocytes. Results showed that β-catenin expression increased with age (p < 0.05, n = 6, Fig. 5A). This finding was consistent with the hypothesis proposed by Deb^17^, but it was against Li’s bioinformatics analysis^18^. To further confirm our results, immunofluorescence and immunohistochemistry were performed, and both showed that the expression of β-catenin increased with age and was widely distributed in the atrial tissues (Fig. 5B,C). These results showed that β-catenin expression is negatively correlated with the proliferative ability of cardiomyocytes, suggesting that β-catenin may also participate in the regulation of young CHD cardiomyocyte cell cycle.

**Figure 6:**
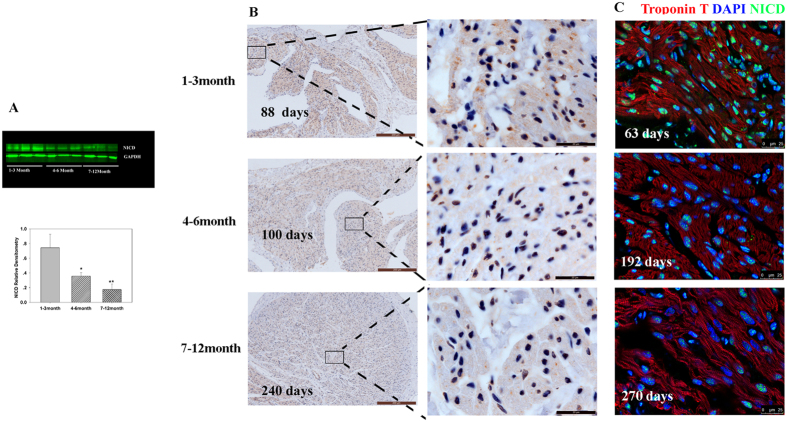
NICD expression in cardiomyocytes. (**A**) Representative Western blot analysis (top) and mean data obtained by densitometry analysis (bottom) are shown. GAPDH served as loading control. The bars indicate mean ± SD. ANOVA was performed to evaluate significance of differences, n = 6, **P < 0.01. (**B**) Representative immunohistochemistry in different age groups. Scale bar: Left panel, 100 μm; Right panel, 20 μm. (**C**) Representative immunofluorescence in different age groups. Scale bar: 25 μm.

Western blot, immunofluorescence, and immunohistochemistry demonstrated that NICD expression declined with age, and NICD was expressed mainly in the nuclei of cardiomyocytes (Fig. 6). Taken together, the expression analysis by multiple methods suggests the regulation of proliferation of young CHD cardiomyocytes may result from the networking of YAP1, β-catenin, and NICD.

## Discussion

Our data showed that the percentage of proliferating cardiomyocytes from 3-month-old hearts is 11 times higher than the percentage in 6-month-old hearts, and 27 times higher than the percentage in 12-month-old hearts (Figs 1 and 2). A recent study demonstrated that between days 0 and 7, the proliferation of mice cardiomyocytes showed diversity: 1-day-old hearts had twice the amount of H3P-positive cardiomyocytes than did 4-day-old hearts. Following stimulation with neuregulin-1, mice cardiomyocytes <7 days old and human myocardial explants <6 months old responded with proliferation, but hearts older than 6 months failed to show proliferation^19^,^20^. Results of aging research suggest that 0–1 month old mice age at a rate that is 150 times faster than that of humans. In certain developmental aspects, 1-month-old mice are comparable to 12-year-old humans^21^. Although no studies confirm the age at which human hearts are comparable to those of 7-day-old mice, current data proved that young CHD cardiomyocytes (≤3 months) have prominent proliferating ability *in vivo* and *in vitro*. This suggests that young CHD cardiomyocytes (≤3 months) may be comparable with cardiomyocytes of ≤3-day-old mice.

Although we have previously shown that there is no significant difference in the number of Ki67-positive cardiomyocytes between normal and VSD hearts^22^, the results should be translated cautiously in healthy human hearts because CHD is a genetically heterogeneous disease, and many proliferation-associated genes have been suggested to play a role in CHD^23^. Therefore, the proliferation rate of healthy human cardiomyocytes may be lesser or higher than that of CHD cardiomyocytes. However, understanding why young CHD hearts keep more proliferation ability in the future will be helpful to comprehend the regulation network of cardiomyocyte proliferation and bring new clues for the heart regeneration study.

It is noteworthy that Ki67-positive cardiomyocytes are relatively smaller in size as shown in Supplemental Fig A and B, suggesting that these proliferating cardiomyocytes may be myocyte-precursors differentiated from embryonic progenitors that have not yet fully specified into cardiomyocytes. These cells may maintain their cell cycle competence, and the progressive decrease in cardiomyocyte cell cycle activity during the first year of life may result from progressive terminal differentiation of cardiomyocytes.

Cell replacement therapy for treatment of a myocardial infarction, particularly after extensive remodeling, will require the introduction of cells under poorly vascularized hypoxic conditions. Under these conditions, adult mature cardiomyocytes are unlikely to survive; however, neonatal immature cardiomyocytes could survive to repopulate damaged tissues^24^. The studies of Reinecke *et al*. reveal that transplanted highly differentiated cardiomyocytes did not form viable grafts and died. However, fetal and neonatal cardiomyocytes survived and proliferated in the impaired myocardium^25^. Our work supports these observations. When cardiomyocytes were isolated from surgical biopsies, the percentages of viable cells (age 1–12 months) in our experiments was 50–70%, but the percentage of viable cells from older patients was around 20% (mean age = 67 ± 8 y)^11^. In addition, few studies have successfully cultured human cardiomyocytes from surgical biopsies^8^,^9^,^10^, and no studies have investigated the proliferation of human cardiomyocytes in culture. As per our knowledge, this is the first study that identified the proliferation maker ki67 and the incorporation of exogenous EdU in cultured pediatric cardiomyocytes.

The regulation of cardiomyocyte proliferation is complex. Animal studies have shown YAP1, β-catenin, and NICD as key factors in their regulation, but at different levels. YAP1 is required for all phases of mouse heart development, from fetus to adult^14^,^16^. β-catenin is involved in multiple phases and activities during cardiac differentiation and development^13^. NICD demonstrates different effects on cardiomyocytes in different species such as zebrafish and mice^26^,^27^. The current study proved that YAP1, β-catenin, and NICD are all correlated with changing CHD cardiomyocyte cell cycle activity. However, during mycoyte specification from cardiac progenitor cells, the level of β-catenin is down–regulated^13^, which is at odds with our data. There are three possible explanations. First, β-catenin signaling can have different effects depending on the time of action^13^, since the first year of life is different from the phase of specification, and Deb hypothesized that β-catenin increases with age and this drives cardiac progenitor cells into the fibrogenic lineage 18). Second, the increased β-catenin level is associated with an increase in cellular senescence and a decrease in population of undifferentiated pluripotent cells^28^. Third, the level of β-catenin in the cytoplasm and nucleus is tightly regulated, and only the nucleus β-catenin functions as a transcription regulator, which is associated with mycoyte specification. In addition, crosstalk between pathways should be studied to investigate why young CHD cardiomyocytes have a higher cell cycle activity.

Our study has shed light on CHD cardiomyocyte proliferation. Some limitations of the study should be taken into consideration. First, data were obtained from atrial tissues, the cell cycle activity of which may be different from ventricular myocytes. Because atrial myocytes are more proliferative than ventricular myocytes^29^ and most of left ventricular myocytes have a different embryonic origin than atrial myocytes^30^, current data cannot be applied directly applied to ventricular myocytes. Second, only YAP1, β-catenin, and NICD expression were investigated. Whether they serve similar roles in CHD patients as in animal models is unknown. Further studies are necessary to determine the mechanisms underlying young CHD cardiomyocyte proliferation.

## Materials and Methods

### Study population and tissue sampling

Right atrial tissues (0.1 cm × 0.1 cm × 0.2 cm) were collected from 111 patients with ventricular septal defects at Shanghai Children’s Medical Center between April 2014 and September 2015. Three samples were used for H&E staining, and the remaining samples were divided into six groups: flowcytometry; laser scanning confocal microscope analysis; cell culture; Western blot; immunofluorescence analysis; and immunohistochemical analysis. Each group included 18 samples and was further divided into three subgroups: 1–3-month group, 4–6-month group, and 7–12-month group. Each subgroup included six samples. All procedures followed the declaration of Helsinki, and were approved by the Animal Welfare and Human Studies Committee at Shanghai Jiaotong University School of Medicine. Parental written informed consent was obtained prior to the initiation of the study.

### Isolation of cardiac cells

After surgical removal, myocardial samples were quickly put in ice-cold aseptic PBS, and transferred to a biosafety cabinet within ten minutes. For cardiac cells isolation, 1-mm^3^ blocks of tissue were washed twice in Solution A: NaCl 120 mmol/L, KCl 5.4 mmol/L, MgSO_4_ 5 mmol/L, pyruvate 5 mmol/L, glucose 20 mmol/L, taurine 20 mmol/L, HEPES 10 mmol/L, and nitrilotriacetic acid 5 mmol/L, pH 7.4. After washing, the tissues were digested by four steps in solution B: NaCl 120 mmol/L, KCl 5.4 mmol/L, MgSO_4_ 5 mmol/L, pyruvate 5 mmol/L, glucose 20 mmol/L, taurine 20 mmol/L, HEPES 10 mmol/L, CaCl_2_ 0.05 mmol/L, and collagenase type II (Sigma, St. Louis, MO, USA), 0.2 mg/mL for 40 minutes. The time for each step was about ten minutes. After each step, dissociated cells were collected in 10% FBS/DMEM-F12 (vol/vol) and gently centrifuged (100× *g*, 4 min, room temperature) to concentrate cardiac cells into a pellet. The washing and digestion steps were carried out at 37 °C in the presence of 95% O_2_ and 5% CO_2_. Washing and digestion solution was pre-oxygenated with 100% O_2_ for at least ten minutes before use.

### Cardiomyocyte Volume Determination

To calculate the cellular volumes of isolated cardiomyocytes, we assumed a cylindrical shape of the cardiomyocyte, if it appeared rod-shaped, or a spherical shape, if it appeared round. Our calculation equation was: Cardiomyocyte Volume = πD^2^L/4(rod shape) orπD^3^/4(round shape). We calculated the cellular volume using Image J (6, Supplemental Figure S1).

### Flowcytometry

Isolated cardiac cells were first fixed with 2% paraformaldehyde/PBS, permeabilized with 0.5% Tween20/(PBS+ 10%FBS) at room temperature for 15 minutes, and stained overnight with mouse monoclonal antibody against cardiac troponin T (Abcam, ab8295, 1:200 dilution) (Abcam, Cambridge, UK) and Alexa Fluor 488–conjugated rabbit anti–Ki67 (Abcam, ab154201, 1:200 dilution). Cardiac cells were washed thrice with PBS, and incubated with Alexa Fluor 647–conjugated anti–mouse second antibody (Abcam, ab150107, 1:1000 dilution). They were washed thrice, and analyzed using a BD FACSAria cell sorter (BD Biosciences, San Jose, CA, USA). Six independent experiments were performed for each analysis.

### Laser scanning confocal microscope analysis for Ki67- and Aurora B -positive cells

For each heart, 150 consecutive cryosections were prepared on 30 slides. Slides were labeled by a random number generator, and every tenth slide was selected for staining and microscopy in a random-systematic fashion. Three researchers, blinded with respect to the samples’ ages and identities, quantified cellular Ki67–positive cells, either by manual count or by digital thresholding (image segmentation and creation of a binary image from a gray scale). Software analysis of the converted binary images was performed with Image Processing and Analysis in Java (Image J).

### Western blotting

Immunoblotting was undertaken to detect the expression of YAP1, β-catenin, and NICD. In brief, individual atrial tissues were solubilized in Laemmli buffer containing 2-mercaptoethanol, and proteins (20 μg/lane) were separated on 10% SDS polyacrylamide gels. After the proteins were transferred onto polyvinylidene fluoride membranes (Merck Millipore, Billerica, MA, USA), the blots were blocked with 5% nonfat milk in Tris-buffered saline with Tween 20 (TBST) for two hours at room temperature, after which they were probed with the following antibodies diluted to 1:1000–1:2500: anti–YAP1 antibody (Ab52771, Abcam), anti–β-catenin antibody (ab32572, Abcam), anti–NICD antibody (Ab83232, Abcam), and anti–GAPDH antibody (ab8245, Abcam). The secondary antibody used was Dylight 800–labeled affinity antibody to rabbit IgG (072-07-15-06 KPL). Quantitative densitometric image analysis was performed (Image J), and the densitometry of the GAPDH band was used for normalization.

### Immunofluorescence

Immunofluorescence was undertaken to confirm the results of Western blot. One hundred and fifty consecutive cryosections were prepared. Labeled slides were numbered with a random number generator, and every fifth slide was picked for staining. The slides were blocked with 10% FBS for 30 minutes, and incubated with the following antibodies diluted to 1:200: rabbit anti–YAP1 antibody (Ab52771, Abcam), rabbit anti–β-catenin antibody (ab32572, Abcam), rabbit anti–NICD antibody (Ab83232, Abcam), rabbit anti-Ki67 antibody(ab15580, Abcam), rabbit anti- Aurora B antibody (ab45145, Abcam), and mouse anti–Troponin T antibody (ab8295, Abcam) at room temperature for two hours. The secondary antibodies used were Fluor 555–conjugated anti–mouse second antibody (Abcam, ab150107, 1:1000 dilution) and Alexa Fluor 488–conjugated anti–rabbit second antibody (Abcam, ab150073, 1:1000 dilution). Nuclear staining was performed with DAPI.

### Immunohistochemistry

To confirm the results of Western blot and immunofluorescence, the other part of the heart tissues was fixed overnight in 4% paraformaldehyde, embedded in paraffin wax, and cut to produce 5-μm–thick sections. For immunohistochemical staining, sections were dewaxed in xylene and rehydrated in serial dilutions of alcohol. Endogenous peroxidase was blocked by immersing the sections in 0.3% H_2_O_2_ in methanol for 20 minutes at room temperature. The specimens were blocked with 3% nonfat milk for two hours at room temperature, and treated using rabbit polyclonal antibodies against Yap1, β-catenin, and NICD at 4 °C for 12–14 hours. Antigen–antibody complexes were detected using a DAB peroxidase substrate kit according to the manufacturer’s protocol (Dako, Carpinteria, CA, USA). The negative controls were subjected to the same protocol except that PBS was used instead of the primary antibody.

### Culture of cardiac cells and immunocytochemistry

For culture, isolated cardiac cells were seeded at a density of 20,000 cells/mL on a 20 μg/mL laminin-pretreated support (Sigma), and cultivated in DMEM/F-12 supplemented with 10% FBS, 1 U/mL Na-Penicillin G, and 0.5 U/mL streptomycin (Gibco, Shanghai, China). In order to inhibit fibroblast proliferation, cultures were performed with 5 μg/ml cytosine □-D-arabinofuranoside (Ara C). Cells were incubated at 37 °C in a humidified, 5% CO_2_–enriched atmosphere. These cells were cultured for four days. To visualize the proliferation of isolated cardiomyocytes, we fixed the cells in 4% paraformaldehyde for ten minutes. After washing, cells were permeabilized with 5% Triton-100 for 15 minutes. The cells were incubated with mouse monoclonal antibody against cardiac troponin T (Abcam, ab10214, 1:200 dilution) and rabbit anti–Ki67 antibody (Abcam, ab15580, 1:200 dilution) at 37 °C for two hours. After washing, cells were incubated with Alexa Fluor 555–conjugated anti–mouse second antibody (Abcam, ab150107, 1:1000 dilution) and Alexa Fluor 488–conjugated anti–rabbit second antibody (Abcam, ab150073, 1:1000 dilution) for 30 minutes. Nuclei were stained with DAPI. For quantification, ten different field pictures were taken from each well, and analyzed using Image J.

### Cell proliferation assay

Cell proliferation was measured by 5-ethynyl-20-deoxyuridine (EdU) incorporation assay, using an EdU assay kit (Ribobio, Guangzhou China), according to the manufacturers’ instructions. Cells were cultured in triplicate in 96-well plates at a density of 1 × 10^4^ cells per well for three days at 37 °C, and then 50 μM of EdU was added to each well and cells were cultured for an additional 24 hours at 37 °C. The cells were fixed with 4% formaldehyde for 15 minutes at room temperature, and treated with 0.5% Triton X-100 for 20 minutes at room temperature for permeabilization. After washing with PBS thrice, 100 μl of 1× Apollo® reaction cocktail was added to each well, and the cells were incubated for 30 min at room temperature. To visualize cardiac myocytes, cells were incubated with anti-sarcomeric alpha actinin antibody (Abcam, ab137346, 1:400 dilution). The cells were stained with 100 μl of DAPI for five minutes, and visualized under a fluorescent microscope (Olympus Corporation, Tokyo, Japan). The EdU-positive and sarcomeric alpha actinin-positive cells were counted using Image-Pro Plus (IPP) 6.0 software (Media Cybernetics, Bethesda, MD, USA). The EdU incorporation rate was expressed as the ratio of EdU-positive and sarcomeric alpha actinin-positive cells to total DAPI-positive cells (blue cells). All experiments were done in triplicate, and three independent repeating experiments were performed.

### Statistical Analysis

Continuous data, including age, cell volume, protein expression, and number of Ki67- and Aurora B-positive cells, were expressed as mean ± standard deviation. Differences were tested by ANOVA. Categorical variables were expressed by count and percentage, which were compared between survival and death by the Fisher’s exact test. P-values < 0.05 were considered statistically significant. Statistical analyses were performed using SAS software version 9.2 (SAS Institute Inc., Cary, NC, USA).

## Additional Information

**How to cite this article**: Ye, L. *et al*. Cardiomyocytes in Young Infants With Congenital Heart Disease: a Three-Month Window of Proliferation. *Sci. Rep*. **6**, 23188; doi: 10.1038/srep23188 (2016).

## Supplementary Material

Supplementary Information
