# Supplementary material for: Cardiomyocytes in Young Infants With Congenital Heart Disease: a Three-Month Window of Proliferation
Source: Sci Rep. 2016 Mar 15;6:23188. doi: 10.1038/srep23188 (PMC4791641; doi:10.1038/srep23188)
Supplement: Supplementary Information [file srep23188-s1.pdf]

Supporting Online Material for

Cardiomyocytes in Young Infants With Congenital Heart Disease: a Three-Month Window of Proliferation

Lincai Ye,[*,†,‡,§] Lisheng Qiu,[*,§] Haibo Zhang,[*] Huiwen Chen,[*] Chuan Jiang,[‡] Haifa Hong,[*,‖] Jinfen Liu,[‡,‖]

[‖]**Address for correspondence:**

Jinfen Liu, Shanghai Institute of Pediatric Congenital Heart Disease, Shanghai Children's Medical Center, Shanghai Jiaotong University School of Medicine, 1678 Dongfang Road, Shanghai 200127, China. Tel: + 86-21-58815377; Fax: + 86 21 50891405; E-mail: jinfenliu2002@163.com.

Haifa Hong, Department of Thoracic and Cardiovascular Surgery, Shanghai Children's Medical Center, Shanghai Jiaotong University School of Medicine, 1678 Dongfang Road, Shanghai 200127, China. Tel: + 86-21-38626649; Fax: + 86 21 50891405; E-mail: hhfsmallboat@163.com.

This PDF file includes:

Figs. S1 to S4

Fig S1

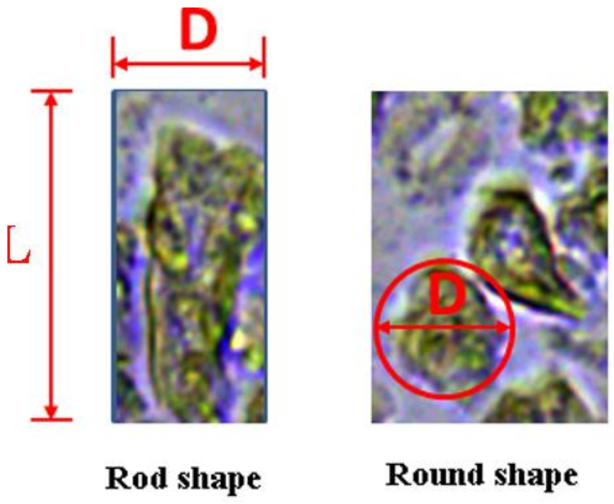

Fig S2

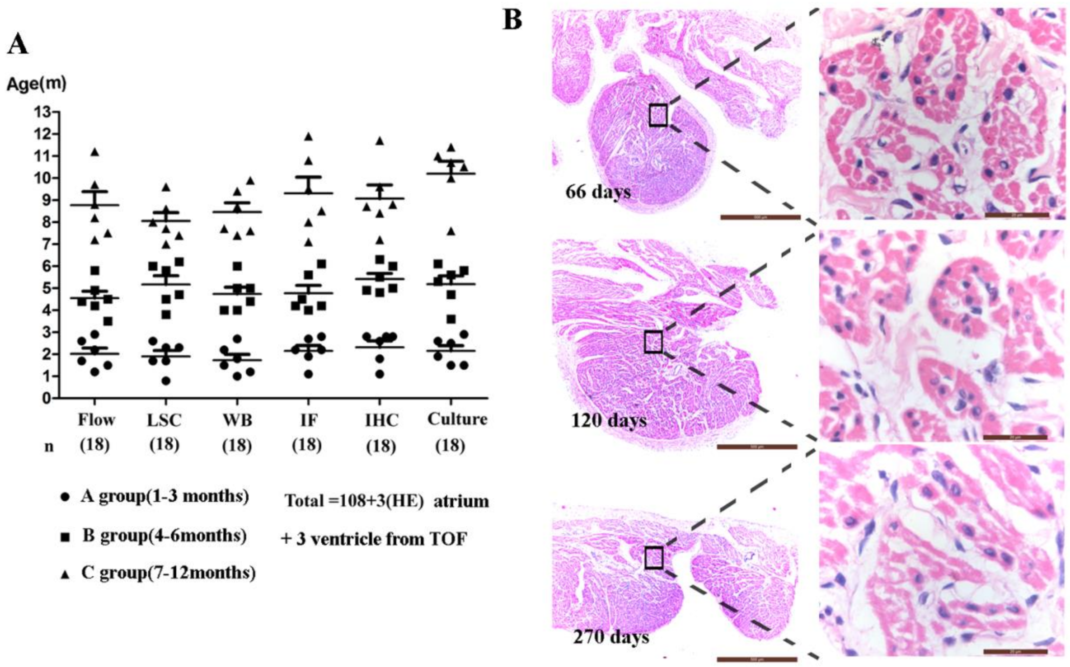

Fig S3

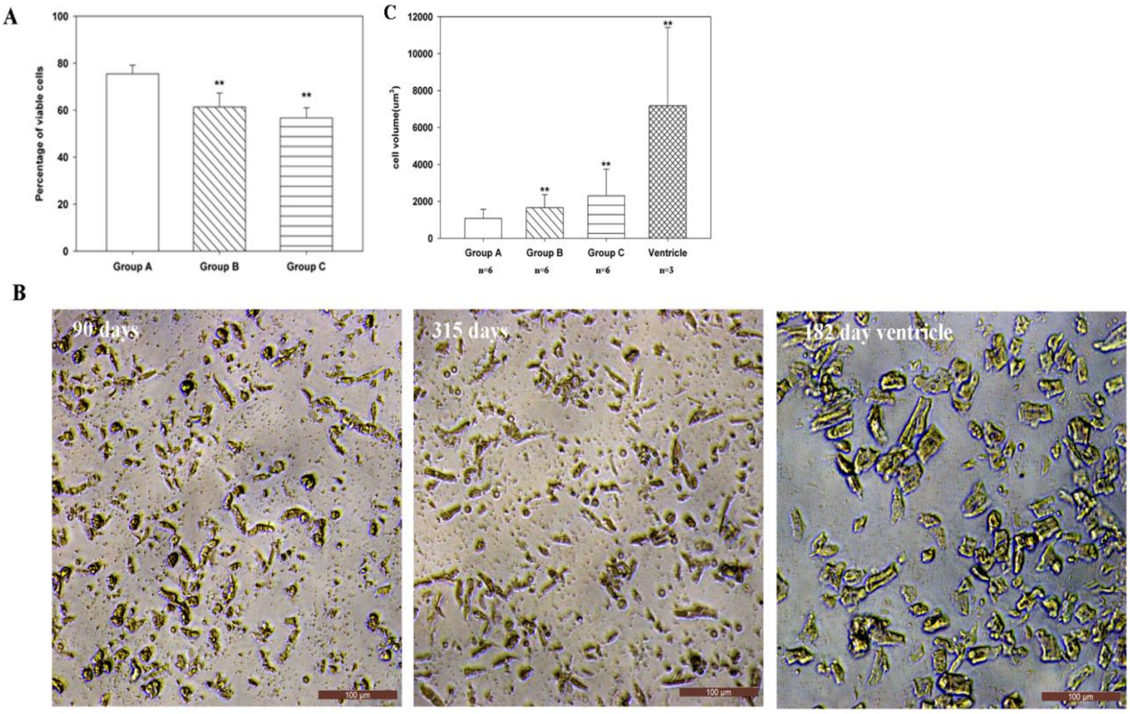

Fig S4

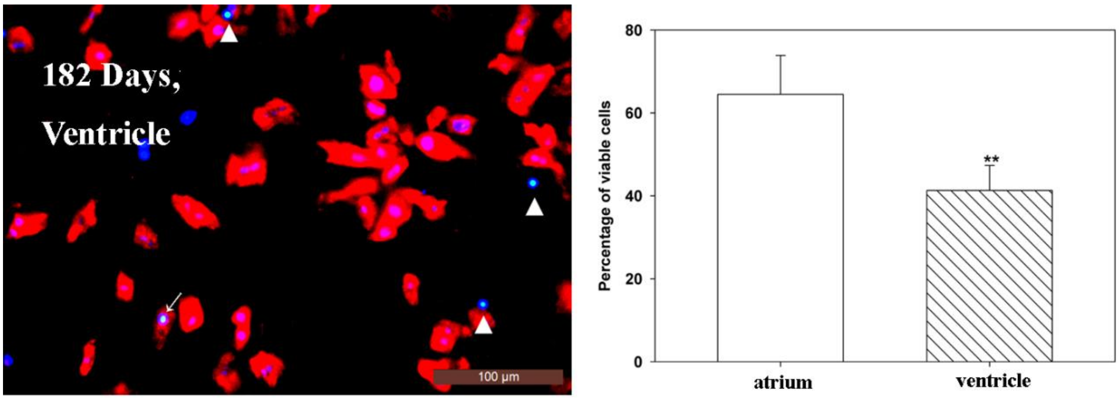

Supplemental fig legends

Fig S1 Method of volume determination for Cardiomyocytes. L = Length, D = Diameter.

Fig S2. (A) Limited tissue sample size allowed for only one method of tissue

assessment in each patient. Out of total 114 patients, 108 tissue samples were distributed into 3 groups according to patient ages, seeking an even age distribution of each group for all the tests; 3 samples were used for HE staining and 3 were from TOF patients. Flow: flow cytometry; LSC: Laser scanning cytometry, for counting Ki67 and Aurora B -positive cardiomyocytes; WB: Western blot analysis; IHC: Immunohistochemistry; IF: immunofluorescence. (B) Histological analysis of atrial tissues. The morphology of atrial tissue from children of different ages was assessed using H&E staining. Scale bars: Left panel = 500 μm; Right panel = 20 μm.

Fig S3. (A) Percentage of viable cells after isolation in different age groups. Ventricle data were from 3 TOF patients. (B) Representative isolated cardiomyocytes from different age groups (C) Cardiomyocyte volumes of different age groups. Cardiomyocytes number in calculated from each patient = 1200, 6 different patients were included in group A, B and C, respectively and 3 different patients were included in group D.

Fig S4. The percentage of viable cells in ventricular cardiomyocytes (182 days) was reduced. (A) Representative ventricular cardiomyocytes. (B) Quantification of viable ventricular cardiomyocytes and atrial cardiomyocytes. Cardiac Troponin T (red), Ki67 (green), and DAPI (blue). Arrow indicates Ki67-positive cardiomyocytes and triangle indicates Ki67-positive non-cardiomyocytes. **$P<0.01$.
